# Ticagrelor induced systemic inflammatory response syndrome

**DOI:** 10.1186/s12872-016-0443-8

**Published:** 2017-01-06

**Authors:** Philipp Krisai, Manuel Haschke, Peter T. Buser, Christian Mueller

**Affiliations:** 1 Department of Medicine, University Hospital Basel, Petersgraben 4, 4031 Basel, Switzerland; 2 Cardiovascular Research Institute Basel, University Hospital Basel, Basel, Switzerland; 3 Division of Clinical Pharmacology and Toxicology, University Hospital Basel, Basel, Switzerland; 4 Department of Cardiology, University Hospital Basel, Basel, Switzerland

**Keywords:** Ticagrelor, SIRS, Case report, Adverse drug reaction

## Abstract

**Background:**

Ticagrelor is a reversible and direct-acting oral antagonist of the adenosine diphosphate receptor P2Y12. Possible adenosine-mediated effects of ticagrelor on inflammation are complex and incompletely understood. To our knowledge, ticagrelor-induced systemic inflammatory response syndrome (SIRS) has not yet been described.

**Case presentation:**

We report the case of an 84 years old patient presenting with SIRS subsequent to initiation of ticagrelor after implantation of two drug eluting stents. A broad diagnostic work-up for alternative causes and therapeutic measures were unrevealing. Discontinuation of the agent was followed by rapid improvement in clinical and laboratory signs of SIRS.

**Conclusions:**

After exclusion of other causes, ticagrelor needs to be considered as a possible causative agent for SIRS. Due to the widespread use of ticagrelor, clinicians should be aware of this possible adverse drug reaction.

## Background

Ticagrelor, a reversible and direct-acting oral antagonist of the adenosine diphosphate receptor P2Y12, significantly reduced death as compared to clopidogrel in patients with acute coronary syndrome [1]. This led to widespread use of the agent and implementation in current guidelines [2]. Possible adenosine-mediated effects of ticagrelor on inflammation are complex and incompletely understood [3]. Due to the lower incidence of sepsis and pulmonary adverse events as well as lower mortality in patients taking ticagrelor versus clopidogrel, such effects were previously considered to be beneficial. To our knowledge, ticagrelor-induced systemic inflammatory response syndrome (SIRS) has not yet been described.

## Case presentation

We present the case of an 84 years old male presenting with dyspnea (NYHA III) and fatigue, hypotension (88/50 mmHg), tachycardia (97 bpm), and fever (38.4 °Celsius) to our emergency department fulfilling 2 of 4 criteria for SIRS [4]. Clinical examination was significant for discrete bibasal pulmonary rales and a 2/6 systolic murmur, in agreement with a preexisting, moderate mitral valve insufficiency. Initial laboratory findings showed substantially elevated C-reactive protein (CRP) (84 mg/l) and serum creatinine (159 μmol/l).

Recent medical history was significant for ST-elevation myocardial infarction (STEMI) 15 days prior to the current presentation with successful percutaneous coronary intervention and implantation of two drug eluting stents in the proximal right coronary artery. Other relevant comorbidities included pre-existing coronary artery disease, arterial hypertension and hypercholesterolemia. His current medication included aspirin, ticagrelor, nebivolol, olmesartan, rosuvastatin and pantoprazol, with ticagrelor initiated 15 days ago. He had no history of allergies.

Empirical antibiotic treatment with ceftriaxone was initiated in the emergency department due to suspected severe sepsis after blood and urine culture sampling. Extensive infectious disease work-up including blood cultures, a respiratory panel for the comprehensive detection of respiratory disease-causing viruses and bacteria, HIV testing, and imaging studies (CT-scan of the chest, abdominal ultrasound, transthoracic and transoesophageal echocardiography) did not reveal an infectious cause of SIRS.

**Fig. 1:**
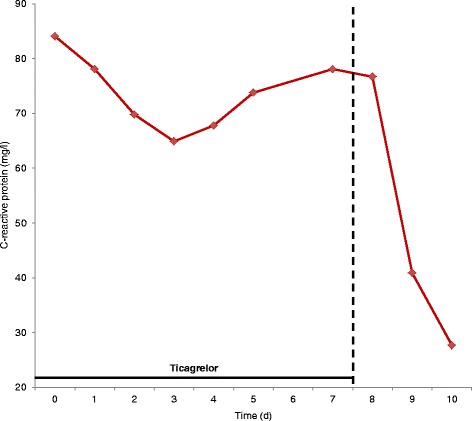
C-reactive protein levels in relation to ticagrelor treatment

Symptoms (dyspnea and fatigue), signs (fever), and laboratory signs (CRP) of SIRS persisted despite 6 days of intravenous antibiotic treatment (Fig. 1). Further, Dressler-Syndrome was considered as a differential diagnosis. However, lack of a pericardial rub, leukocytosis, pericardial effusion, or clinical and laboratory response to preemptive treatment with ibuprofen rendered it very unlikely.

After broad, unrevealing diagnostic work-up ticagrelor was suspected as the causative agent of persistent SIRS due to recent initiation and no other change in drug treatment. Accordingly, ticagrelor was discontinued at day seven and replaced by clopidogrel. No other drug was changed during the hospital stay. This was followed by a rapid improvement in symptoms as well as clinical and laboratory signs of SIRS. In clinical follow-up 2 weeks and 1 year after discharge the patient remained asymptomatic and well.

## Discussion

To our knowledge this is the first report describing ticagrelor induced SIRS. The close temporal association between the initiation and discontinuation of ticagrelor with the onset and resolution of SIRS, as well as the lack of evidence for an alternative cause despite extensive diagnostic and therapeutic measures justify considering a causal relationship as possible. Two unlikely differential diagnoses remain. First, the patient could have suffered from sepsis due to viral infection which escaped detection despite the broad diagnostic work-up and by chance resolved exactly at the time of discontinuation of ticagrelor. Second, the patient could have suffered from a self-limiting non-infectious inflammatory disease without any additional rheumatologic symptoms and/or signs, which by chance started soon after the initiation of ticagrelor and also by chance resolved exactly at the time of discontinuation of ticagrelor.

A recent study showed discontinuation of ticagrelor in 17% of treated patients. Conventional side effects of ticagrelor included dyspnea, bleeding, dizziness, rash, itching and gastrointestinal adverse reactions. However the most frequent cause of discontinuation was the need for oral anticoagulation therapy [5]. Additionally frequent comorbidities in patients with ischemic heart disease like chronic obstructive pulmonary disease may influence discontinuation rates of ticagrelor. This is mainly due to known side effects including dyspnea and bleeding [6]. However evidence is missing to suggest a different use of ticagrelor in this patient population [7].

Interactions between platelet P2Y12 inhibitors and the immune system result in both favorable, including reduced incidence of sepsis and pulmonary adverse events, and adverse, including elevation of CRP and dyspnea, effects [1, 3]. Underlying mechanisms are not fully understood. Potential mechanisms are inhibition of leukocyte-platelet interactions with alterations in more downstream inflammatory processes, the inhibition of P2Y12 receptors on other cells, including dendritic and vascular smooth muscle cells, and an increase in extracellular adenosine [3]. Adenosine acts at low concentrations pro-inflammatory via activation of leukocyte A_1_ receptors. Thereby chemotaxis of neutrophils and phagocytosis of neutrophils and macrophages are facilitated. However, at higher concentrations mostly leukocyte A_2_ receptors are activated with subsequent decreased release of pro-inflammatory cytokines. Ticagrelor-induced changes in adenosine levels may therefore induce an imbalance in this complex system [3].

It is therefore important to consider ticagrelor as a causative agent in a patient presenting with SIRS soon after the initiation of ticagrelor. Further research is urgently needed to better elucidate the interactions between ticagrelor and the immune system considering the widespread use of the drug.

## Conclusions

Ticagrelor needs to be considered as a possible causative agent in patients presenting with SIRS whenever the diagnostic work-up for infectious causes is unrevealing.

## Abbreviations

CRP: C-reactive protein
SIRS: Systemic inflammatory response syndrome
